# Atypical Case of Schmidt’s Syndrome in a Young Male

**DOI:** 10.7759/cureus.26322

**Published:** 2022-06-25

**Authors:** Ikram Abdullah, Abdullah S Bdaiwi, Majd Wess, Robert R Camferdam

**Affiliations:** 1 Internal Medicine, Arkansas College of Osteopathic Medicine, Fort Smith, USA; 2 Biomedical Engineering, University of Cincinnati, Cincinnati, USA; 3 Internal Medicine, Unity Health, Searcy, USA; 4 Nephrology, Unity Health, Searcy, USA

**Keywords:** autoimmune, schmidt’s syndrome, autoimmune hypothyroidism, type 1 diabetes, addison’s disease

## Abstract

Autoimmune polyendocrine syndrome type 2, also known as Schmidt’s syndrome, is a rare autosomal dominant life-threatening syndrome. It is defined by the presence of Addison’s disease in combination with at least one of the known autoimmune diseases: thyroid autoimmune disease, type 1 diabetes, and hypogonadism. It is more common in middle-aged females and is treatable if diagnosed early. However, in this case, we report Schmidt’s syndrome in a young male without a family history.

A 20-year-old male with a past medical history of hypothyroidism, adrenal insufficiency, and type 1 diabetes presented to the emergency department (ED) feeling lethargic, somnolent, and diaphoretic. Laboratory blood tests showed elevated thyroid-stimulating hormone, hyperkalemia of 6.4 mmol/L, and hyponatremia of 131 mmol/l indicating an Addisonian crisis. The patient had low blood glucose (at home: 60 mg/dL, and at ED: 85 mg/dL), hypotensive blood pressure of approximately 85/55 mmHg, and a peaked T-wave on EKG, which were consistent with the diagnosis of Schmidt’s syndrome.

Based on the laboratory findings and history, the patient was diagnosed with polyendocrine syndrome Type 2 (Schmidt’s syndrome). The patient was treated for adrenal insufficiency first followed by thyroid insufficiency.

Schmidt’s syndrome is a rare disease and difficult to diagnose because the presentation depends on which gland is initially involved. A few cases have been reported in the literature of atypical presentations of Schmidt’s syndrome. Therefore, this case report can contribute to the medical literature on Schmidt’s syndrome, which can help in early diagnosis and improve patient outcomes.

## Introduction

Schmidt’s syndrome is a rare autosomal dominant disease that is more common in middle-aged females with a prevalence of 1.4 to 4.5 per 100,000 population [1-6]. It is defined by the presence of Addison’s disease in combination with at least one of the known autoimmune diseases: thyroid autoimmune disease, type 1 diabetes, and primary hypogonadism [3-5]. Schmidt’s syndrome is a life-threatening syndrome if not diagnosed and treated early. It is typically treated by separately treating each of its component’s diseases with hormonal replacement therapy [1,3-5].

Here, we report a unique patient with Schmidt’s syndrome, usually inherited in an autosomal dominant pattern. However, this patient is a young male with no family history of Schmidt’s syndrome. The presented patient has adrenal insufficiency, type 1 diabetes, and permanent hypothyroidism without non-endocrine manifestations. This disease combination is rare; only a few cases with similar presentations have been reported in young males.

## Case presentation

Written consent was obtained from the patient. A 20-year-old Caucasian male presented to the emergency department (ED) with the chief complaint of feeling lethargic, somnolent, diaphoretic, and answering only with a “yes” or “no.” The patient had a past medical history significant for hypothyroidism, type 1 diabetes, and Addison's disease. The patient was previously seen and hospitalized for Addisonian crisis due to medication noncompliance. The patient was given steroids to stabilize and then discharged after reaching his baseline. He was also recommended to follow up with an endocrinologist.

After about seven months, the patient came back to the ED with fatigue, weakness, shaking, mental status change, lethargy, and occasional dyspnea with walking. The patient stated that he had an abnormal sleeping schedule for several weeks and mostly stayed up late at night while sleeping in the morning. As a result, he forgot to take his daily medications quite frequently. His medications included fludrocortisone acetate (0.1 mg, every other day), hydrocortisone (10 mg twice daily at 8 am and 2 pm), and levothyroxine (75 mcg once daily). During the initial physical exam, the patient appeared confused, and sluggish to respond to questions. He had a blood pressure of 85/55 mmHg, a pulse of 58 beats/min, a temperature of 95.1°F, and a BMI of 19.37. The patient was alert, awake, and oriented to self, time, and place. He had dry mucus membranes. A neck examination revealed a supple neck without jugular venous distention, bruits, adenopathy, or palpable masses. Cardiovascular examination revealed regular rate and rhythm, and normal S1 and S2 heart sounds without murmur, rub, gallop, or thrills. Lungs were clear to auscultation and percussion. Abdominal examination revealed normal bowel sounds without tenderness or organomegaly and an insulin pump in the left lower quadrant. Neurological examination revealed normal cranial nerve 2-12 with bilateral upper and lower extremity muscle weakness. Glasgow coma score was 14 (eye-opening (4)-spontaneous, verbal (4)-confused/disoriented, motor (6)-obeys commands/spontaneous). His skin was cool, pale, and clammy with increased diffuse pigmentation most notable in the lips. The rest of the physical exam was unremarkable.

The patient was admitted to the hospital for low blood glucose (at home: 60, and at ED 85 mg/dL), hypotension, peaked T-wave on EKG, hyperkalemia of 6.4 mmol/L, and hyponatremia of 131 mmol/L indicating Addisonian crisis. Given the patient’s clinical condition in the emergency room, he was immediately placed on Solu-Cortef (hydrocortisone sodium succinate) 50 milligrams IV push x2 and a 1-liter bolus of normal saline x1. Additionally, he was given calcium gluconate (IV infusion) to stabilize his myocardium; and insulin, dextrose, and Lokelma (sodium zirconium cyclosilicate) to reduce his potassium level while in the intensive care unit. The patient improved symptomatically with the treatment over two days, and his peaked T-wave on EKG was resolved. The patient was restarted on hydrocortisone, fludrocortisone, and levothyroxine. The patient was stable for transfer to the inpatient unit. The patient had been controlling his diabetes via a continuous glucose monitor and a connected insulin pump. At the time of his discharge, the patient's symptoms improved, and he reported to be back to his normal baseline. Based on the history and laboratory findings (see Table 1), the patient was later diagnosed with autoimmune polyendocrine syndrome type 2 (Schmidt’s syndrome) without non-endocrine autoimmune diseases.

**Table 1:** Patient laboratory data on administration. ^1^BUN=Blood Urea Nitrogen, ^2^SGOT/AST=serum glutamic oxaloacetic transaminase/aspartate aminotransferase, ^3^SGPT/ALT=serum glutamic pyruvic transaminase/Alanine aminotransferase, ^4^TSH=thyroid-stimulating hormone, ^5^WBC=white blood cell count, ^6^RBC=red blood cell count, ^7^HGB=Hemoglobin, ^8^HCT=hematocrit, ^9^MCV=mean corpuscular volume, ^10^MCHC=mean corpuscular hemoglobin, ^11^MCHC=mean corpuscular hemoglobin concentration, ^12^RDW=red cell distribution width, ^13^MPV=mean platelet volume, ^14^PLT=Platelets, ^15^NEUT=Neutrophil, ^16^LYMP=Lymphocyte, ^17^MONO=monocyte, ^18^EOS=eosinophil, ^19^BASO=basophil, ^20^IG=immunoglobulin, ^21^ANC=absolute neutrophil count.

	Value	Normal Range		Value	Normal Range
Sodium	131	137-145 mmol/L	^7^HGB	13.6	13.5-18.0 g/dL
Potassium	6.4	3.5-5 mmol/L	^8^HCT	40.6	40.0-54.0%
Chloride	102	98-107 mmol/L	^9^MCV	84	80-94 fL
CO2	21	22-30 mmol/L	^10^MCH	28.2	27.0-31.0 pg
Anion gap	8	5-15 mmol/L	^11^MCHC	33.5	33.0-37.0 g/dL
Glucose	121	75-110 mg/dL	^12^RDW	11.9	11.5-14.5%
^1^BUN	21	9-20 mg/dL	^13^MPV	11.8	7.4-10.4
Creatinine	0.8	0.8-1.5 mg/dL	^14^PLT	264	150-450 th/µL
Calcium	9.8	8.4-10.2 mg/dL	^15^NEUT%	35.6	40.0-80.0%
T Bilirubin	0.4	0.1-1.3 mg/dL	^16^LYMP%	40.8	20.0-40.0%
Alkaline Phos	66	38-126 U/L	^17^MONO%	14.6	0.0-10.0%
^2^SGOT/AST	43	5-34 U/L	^18^EOS%	8.2	0.0-5.0%
^3^SGPT/ALT	35	11-55 U/L	^19^BASO%	0.6	0.0-2.0%
Total protein	7.3	6.3-8.2 g/dL	^20^IG%	0.2	0.0-1.0%
Albumin	4.4	3.2-5.0 g/dL	^21^ANC	1.9	
^4^TSH	5.62	0.46-4.68 mlU/L	Influenza A	Negative	
^5^WBC	5.2	4.8-10.8 th/µL	Influenza B	Negative	
^6^RBC	4.82	4.70-6.10 mil/µL	SARS-CoV-2	Negative	

## Discussion

Autoimmune polyendocrine syndromes (APSs) are rare autoimmune disorders that are characterized by functional impairment of multiple endocrine glands due to loss of immune tolerance. Moreover, these syndromes can present with non-endocrine autoimmune diseases such as alopecia, vitiligo, celiac sprue, and pernicious anemia [2,4,5]. APS is usually categorized into either a rare monogenic form called APS type 1 (APS-1), or a more common polygenic form called polyendocrine syndrome type 2 (APS-2), Schmidt’s syndrome [1,2,4,5].

APS-1, known as APECED (autoimmune polyendocrinopathy-candidiasis-ectodermal dystrophy), MEDAC (multiple endocrine deficiency autoimmune candidiasis syndrome), and juvenile autoimmune polyendocrinopathy, is a rare autosomal recessive disease caused by mutations in the autoimmune regulator gene (AIRE) where the prevalence is estimated to be 1:80,000 in most countries [3]. APS-1 is defined by the combination of at least two of three cardinal component diseases including chronic mucocutaneous candidiasis, hypoparathyroidism, and primary adrenal insufficiency (Addison’s disease). It is more prevalent in infancy, and often reports yeast infection of the mouth and nails as a first visible sign [1,2,5].

APS type 2 (APS-2), Schmidt’s syndrome is defined by the presence of Addison’s disease in combination with at least one of the following autoimmune diseases: thyroid autoimmune disease, type 1 diabetes, and primary hypogonadism [3-5]. This condition has different disease presentations and exhibits familial aggregation. The mechanism of Schmidt’s syndrome cause is not well known. However, it is often observed following an abnormal immune response. It has been found that Schmidt’s syndrome is associated with major histocompatibility complex type 2 specifically DR3-DQ2 and DR4-DQ8. Consequently, Schmidt’s syndrome has been linked to celiac disease, type 1 diabetes, autoimmune thyroid disease, and Addison’s disease [1-4].

The thyroid autoimmune condition linked to Schmidt's syndrome might manifest as Graves' disease or chronic autoimmune thyroiditis (Hashimoto's thyroiditis). However, Hashimoto’s thyroiditis is more common in individuals with Schmidt’s syndrome. In these cases, attempting to restore normal thyroid function may result in adrenal failure or what is known as an Addisonian crisis. Therefore, clinicians must pay extra attention and consider the course of the treatment plan [2]. Schmidt’s syndrome can also be associated with other diseases such as celiac disease, vitiligo, pernicious anemia, myasthenia gravis, alopecia, hypergonadotropic hypogonadism, and hypophysitis [3].

Upon reviewing the present medical literature, we learned that Bhullar et al. [1] reported an African male (25 years) who had hypothyroidism, hypopituitarism, and adrenal insufficiency in his medical history. The patient was admitted with weakness, weight loss, confusion, and sporadic “fainting” spells for a few weeks. A physical examination revealed a heart rate of 56 beats per minute and blood pressure of 122/71 mmHg. Vitiligo on digits and flat affect were also noted. Initial laboratory results were significant for total T3 (23 ng/dL), free T4 (<0.20 ng/dL), TSH (58.18 µIU/mL), testosterone levels (226), and ACTH (less than 2). According to the patient's medical history and tests result, the patient was diagnosed with primary adrenal insufficiency, autoimmune hypothyroidism, hypogonadism, and autoimmune hypophysitis with growth hormone deficiency. This patient was diagnosed with Schmidt’s syndrome involving four glands: adrenal, thyroid, pituitary, and gonads. Additionally, the patient had a non-endocrine manifestation of Schmidt’s syndrome, vitiligo.

Gumieniak et al. [7] also reported a 31-year-old man who presented with nausea, anorexia, and weakness for six days. The patient had a lack of energy, increased constipation, and easy tanning several weeks before admission. His physical exam in the ED showed low blood pressure of 100/60 mmHg, a pulse of 84 beats/min, mild periorbital edema, dry mucus membranes, small, non-tender goiter, and increased diffuse pigmentation of the skin. Initial laboratory showed severe hyponatremia of 101 mEq/L and hyperkalemia. The Cosyntropin stimulation test showed a low baseline cortisol level and high corticotropin level confirming the diagnosis of primary adrenal insufficiency. Moreover, a computed tomographic scan displayed bilateral adrenal atrophy, affirming the diagnosis of autoimmune adrenal failure. The patient was diagnosed with severe primary hypothyroidism. This patient was diagnosed with Schmidt’s syndrome involving only two endocrine glands: thyroid and adrenal glands.

Even though Schmidt’s syndrome has been reported multiple times in the medical literature, our case report is unique because our patient is a young male with no family history of Schmidt's syndrome. Nonetheless, Schmidt’s syndrome inheritance pattern is autosomal dominant and more common in middle-aged females. Our patient was diagnosed with Schmidt’s syndrome encompassing adrenal insufficiency, autoimmune thyroiditis, type 1 diabetes, and without non-endocrine manifestations. This presentation of the disease is rare and atypical, which would serve as a guide for physicians to help diagnose and treat Schmidt’s syndrome early. 

## Conclusions

Schmidt's syndrome can be a life-threatening syndrome if not diagnosed early and treated. It also can be misdiagnosed due to its similar early symptoms to a wide range of other illnesses and diseases. Thus, we advise physicians carry higher suspicion of Schmidt’s syndrome in patients with idiopathic endocrine deficiencies as their early treatment with hormonal replacement therapy can be lifesaving.
